# Severe congenital ocular coloboma

**DOI:** 10.11604/pamj.2014.19.1.5025

**Published:** 2014-09-01

**Authors:** Samar Younes, Hicham Tahri

**Affiliations:** 1 Ophthalmology Service, CHU Hassan II, Fez, Morocco

**Keywords:** Ocular coloboma, congenital, embryonal fissure

## Image in medicine

Congenital ocular colobomas are the result of a failure in closure of the embryonal fissure. They are important causes of childhood visual impairment and blindness. A 22 year old female patient with no particular history complaining of blurred vision of left eye; Visual acuity of the left eye is limited to counting finger; examination of the anterior segment was unremarkable. At fundoscopy, a large coloboma involving the optic disc and the adjacent retina. Examination of the right eye was normal. General examination was unremarkable including the neurological examination. Ocular coloboma can be seen in isolation and in an impressive number of multisystem syndromes. Systemic associations include the CHARGE syndrome (Coloboma, heart defects, atresia of the choane, retardation, genital defects and ear defects). Visual acuity can range from normal to severly impaired. In general, severity of disease can be linked to the temporalexpression of the gene, but this is modified by factors such as tissue specificity of gene expression and genetic redundancy.

**Figure 1:**
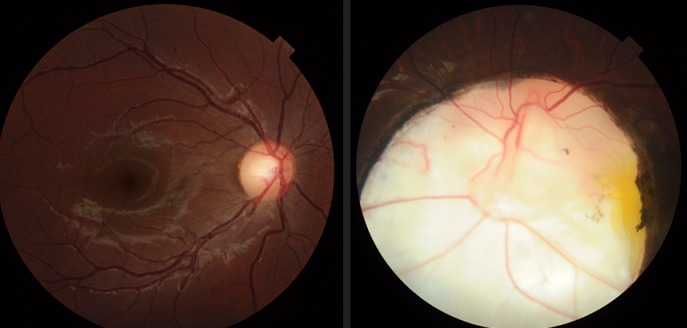
Congenital ocular coloboma

